# Symptoms of Premenstrual Syndrome in Women Receiving Seed Cycling Capsules With Chasteberry: A Single-Arm Clinical Trial Over Two Cycles

**DOI:** 10.7759/cureus.115036

**Published:** 2026-08-23

**Authors:** Ellen O'Gorman, Ulrike W Kaunzner, Susanne Mitschke

**Affiliations:** 1 Clinical Research, Citruslabs, Las Vegas, USA; 2 Clinical Research, Two Moons Health, New York, USA

**Keywords:** dietary supplement, estrogen, menstrual cycle, pms, premenstrual syndrome, progesterone, seed cycling

## Abstract

Premenstrual syndrome (PMS) and menstrual irregularities are common concerns among women in their menstruating years and may negatively affect physical health, emotional well-being, daily functioning, and overall quality of life. This prospective, virtual, single-arm clinical trial evaluated the use of Two Moons Seed Cycling Capsules, ZEST and ZEN, on changes in the severity and frequency of PMS-related symptoms across two menstrual cycles. ZEST, comprising organic flaxseed and organic pumpkin seed, was taken during days 1-14 of the cycle, while ZEN, comprising organic sesame seed, organic sunflower seed, and other organic ingredients not traditionally used in seed cycling, psyllium, and chasteberry (also known as *Vitex agnus-castus*), was taken during days 15-28 of the cycle. A total of 40 women aged 21-46 years completed a baseline survey and began product use at the start of their next menstrual period. Follow-up questionnaires were completed at the end of Cycle 1 and Cycle 2. By the end of Cycle 2, participants demonstrated significant improvement in Menstrual Symptom Questionnaire (MSQ) scores, Numerical Pain Rating Scale (NPRS) scores for menstrual cramping, and a 73.86% decrease in the number of sick days attributed to menstrual-related issues. All nine evaluated PMS-related symptom parameters showed significant improvement by the end of Cycle 2. These included reductions in the frequency of digestive upset, breast tenderness, and PMS symptoms that interfered with productivity. Participant perception data further indicated strong satisfaction with the product experience. By the end of Cycle 2, 10 of 15 product-related statements received strong (≥70%) or majority agreement (50-69%) from the participants, including perceived improvements in premenstrual and menstrual pain (n = 28, 71.79%), cramping (n = 26, 66.67%), bloating (n = 26, 66.67%), mood (n = 24, 61.54%), sleep (n = 24, 61.54%), and skin clarity (n = 24, 61.54%). Overall, the findings suggest that intake of the test product was associated with cumulative reductions in the severity and frequency of PMS-related symptoms over two menstrual cycles. These results support the potential role of seed cycling capsules as a wellness intervention for managing premenstrual and menstrual symptoms. However, because this study used an open-label, single-arm design without a control group, further large-scale, randomized, controlled studies are recommended to validate these findings and better assess the efficacy of the test product.

## Introduction

Premenstrual syndrome (PMS) is a condition affecting approximately 47.8% of women globally [1]. The condition is characterized by a recurring pattern of physical, emotional, or behavioral symptoms that occur in the five days before menstruation, are present for at least three consecutive menstrual cycles, resolve within four days after menstruation begins, and interfere with normal activities. Symptoms appear during the luteal phase and subside after menstruation begins and may include bloating, headaches, anxiety, mood swings, poor concentration, and sleep disturbances. For many women, PMS has a profound effect on quality of life, as the myriad of symptoms impact mental health, occupational obligations, social activities, and sleep, changes that also exacerbate symptom severity and contribute to a cyclical deterioration in overall well-being. The underlying cause of PMS has not yet been defined, but it is thought to involve heightened sensitivity to normal cyclical changes in ovarian hormones after ovulation, particularly progesterone, as well as estrogen-related effects [2].

Current treatments for PMS address mood symptoms through selective serotonin reuptake inhibitors (SSRIs) or stabilize hormonal fluctuations through the use of contraceptives. However, these options do not always address underlying hormonal abnormalities, may cause adverse effects that further constrain quality of life, or place a financial burden on users. To address these concerns, seed cycling could provide a low-cost, well-tolerated, and potentially effective adjunct for managing PMS. Seed cycling is a dietary approach in which specific seeds are consumed at different stages of the menstrual cycle, typically flaxseed and pumpkin seeds during the follicular phase, followed by sesame and sunflower seeds during the luteal phase, with the goal of supporting hormonal balance [3].

The rationale for seed cycling is based on the nutritional profile and underlying biological influences of selected plants and seeds that can impact hormonal balance. Flaxseeds provide a natural source of lignans and omega-3 that can modulate estrogen via phytoestrogenic activity and improve the progesterone-to-estrogen ratio [3-6]. Similarly, the zinc content of pumpkin seeds can support follicle-stimulating hormone (FSH) and luteinizing hormone (LH) synthesis, thereby promoting ovulation [3]. Sesame seeds contain concentrated levels of lignans and phytoestrogens that may modulate estrogen receptor activity and improve the progesterone-to-estrogen ratio [3]. Chasteberry (*Vitex agnus-castus*), though not traditionally included in seed cycling, may similarly support luteal-phase progesterone levels through a distinct mechanism; chasteberry contains diterpenes that act on dopamine D2 receptors in the pituitary, reducing prolactin secretion. In some clinical studies, this has been associated with normalization of luteal-phase function and increased mid-luteal progesterone, suggesting an effect on hypothalamic-pituitary-ovarian regulation [7-9]. Preclinical data also suggest broader endocrine modulation, including possible effects on pituitary-adrenal signaling, although human evidence for a direct hypothalamic-pituitary-adrenal axis effect remains limited [10]. Rich in vitamin E and selenium, sunflower seeds may support progesterone synthesis [3,11,12]. Other fiber-rich ingredients may also contribute to hormonal balance by binding intestinal estrogens, reducing β-glucuronidase, and raising sex hormone-binding globulin [13] and may support broader symptom management. For example, psyllium exhibits antioxidant and anti-inflammatory properties in gastrointestinal disease, effects that may also help to reduce inflammation-related menstrual pain [14].

Empirical evidence for seed cycling in the treatment of PMS is emerging. Across two randomized controlled trials, intake of chasteberry has been demonstrated to improve responses to self-assessed questionnaires, including the Premenstrual Tension Scale (PMTS), in women with PMS compared with placebo [15,16]. Intake of flaxseed has also been shown to lengthen the luteal phase by a mean of 1.2 days and significantly raise the progesterone-to-estradiol (P/E2) ratio in normally cycling women [5], while intake of flaxseed together with sesame seeds, sunflower seeds, and pumpkin seeds has been shown to improve menstrual cycle regularity and hormonal balance in women with polyendocrine metabolic ovarian syndrome (PMOS), previously named polycystic ovary syndrome (PCOS), across clinical trials and case studies [4,17]. Although direct evidence evaluating seed cycling for PMS remains limited, these findings provide preliminary support for the potential hormonal and symptomatic benefits of seed-based interventions.

In light of these preliminary findings and the current lack of direct evidence evaluating structured seed cycling protocols, the present study was designed to investigate a seed cycling-based intervention for the management of PMS-related symptoms. The Two Moons Seed Cycling Capsule product comprised two phase-specific formulations: ZEST, containing organic flaxseed and pumpkin seed, taken during days 1-14 of the cycle, and ZEN, containing organic sesame seed, sunflower seed, chasteberry, and psyllium, taken during days 15-28. All participants adhered to the schedule regardless of when their second period started. The product also included psyllium to target gastrointestinal discomfort, inflammation-related symptoms, and broader PMS symptom relief. A single-arm, virtual, two-cycle study design was selected to generate preliminary real-world data while minimizing participant burden and enabling repeated assessment over two menstrual cycles. Through this approach, the study aimed to evaluate changes in PMS-related symptom severity and frequency associated with use of the test product.

## Materials and methods

Study design and setting

This was a prospective, virtual, open-label, single-arm interventional study conducted over two menstrual cycles between July and September 2025. The study was sponsored by Two Moons Health. Citruslabs served as the clinical research organization and provided clinical research services for the trial. The study was designed to evaluate changes in PMS-related symptom frequency and severity associated with use of the Two Moons Seed Cycling Capsules with chasteberry, as analyzed through self-reported outcomes and standardized assessments of PMS-related symptoms. The study was carried out remotely, with all screening, informed consent, product use, and outcome assessments completed virtually. Participants completed questionnaires via an online study platform at Baseline, at the end of their first cycle (Cycle 1), and at the end of their second cycle (Cycle 2) (Appendices). The total duration of the study for each participant was the completion of two menstrual cycles. A placebo or comparator group was not included. All analyses evaluated within-participant changes at each time point from Baseline.

Ethics approval and informed consent

The study protocol was approved by the Independent Review Board (IRB), Elemental IRB, on June 3, 2025, under approval number 20756, and the trial was registered prospectively with ClinicalTrials.gov (NCT: NCT07655466). All participants provided electronic informed consent before any study-specific procedures were carried out. The study was conducted in accordance with the ethical principles of the Declaration of Helsinki.

Participant selection and eligibility criteria

Participants residing in the United States were recruited by Citruslabs using its participant database. A total of 330 participants were assessed for eligibility, and 40 participants were enrolled. A target sample size of 40 participants was selected to provide preliminary estimates of within-participant changes in PMS-related outcomes over two menstrual cycles while maintaining feasibility for remote recruitment, study administration, and follow-up. The sample size was not based on a formal hypothesis-driven power calculation, as the study was intended to generate preliminary data to inform future randomized controlled trials.

Adults aged 18-45 years with a regular menstrual cycle between 23 and 35 days were eligible to participate in this study if they tracked their menstrual cycle and had self-reported concerns with five or more of the following symptoms in the last three menstrual cycles: heavy flow, prolonged, shortened, or irregular periods, headaches or migraines, low mood, fatigue, bloating, premenstrual or menstrual cramps, breast tenderness, poor sleep, constipation or diarrhea, weight gain or weight fluctuations, irritability or anger, sick days or reduced social time due to menstrual symptoms, or hormonal acne. Participants were required to refrain from taking any other products, medications, or supplements targeting menstrual health for the duration of the trial and to complete all questionnaires electronically.

Participants were excluded from the study if they were in menopause or were experiencing menopausal-type symptoms, were currently taking hormonal contraception, had a planned insertion or replacement of an IUD in the next 12 months, or were pregnant, breastfeeding, or trying to conceive within the next two months. Participants were also excluded if they had any chronic conditions that would prevent adherence to the protocol, including cancer and mental health disorders other than menstrual-related dysphoria or depression; had allergies or sensitivities to any of the product ingredients; had a history of substance abuse; had diagnosed diabetes, an endocrine-related condition, or other endocrine disorders (i.e., endometriosis, adenomyosis, or PCOS); or were taking hormone-modulating medication, such as hormone replacement therapy, or any other hormone-altering therapy. Additionally, participants who had planned surgery during the study period, were participating or planned to participate in another research study during the study period, or were unwilling to follow the study protocol were excluded.

Test product and intervention

All participants received the intervention comprising ZEST and ZEN capsules. Participants were to wait until the beginning of their next period before using the test products as directed, taking two capsules of ZEST or ZEN every morning with the first meal of the day. The ZEST capsule, taken from days 1-14 of the menstrual cycle, contained organic flaxseed and organic pumpkin seed. ZEN, taken from days 15-28 of the menstrual cycle, contained organic sesame seed, organic sunflower seed, organic psyllium, and organic chasteberry (Table 1). All participants adhered to the schedule regardless of when their second period started, spending 14 days on ZEST, then 14 days on ZEN, then another 14 days on ZEST, and finally another 14 days on ZEN.

**Table 1:** Ingredients of ZEST and ZEN seed cycling capsules.

Formulation	Component	Dose (per 2 capsules)
ZEST	Organic flaxseed	800 mg
	Organic pumpkin seed	650 mg
ZEN	Organic sesame seed	800 mg
	Organic sunflower seed	550 mg
	Organic psyllium	80 mg
	Organic chasteberry	20 mg

Study procedures and assessment

**Figure 1:**
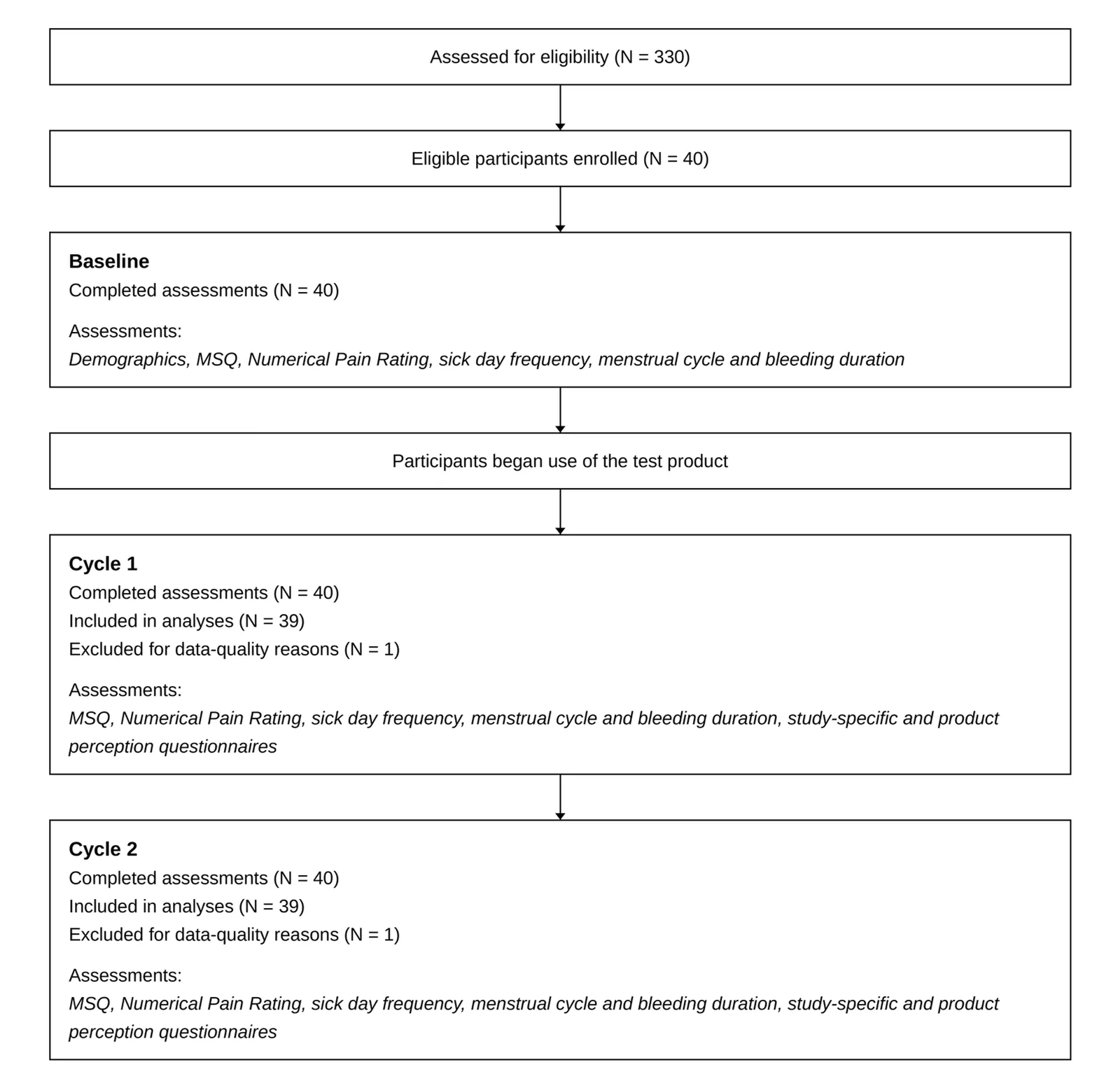
Study assessment schedule. A total of 40 participants were eligible and enrolled in the study. Participants who failed quality-control questions at individual time points were excluded from analyses for those time points only. MSQ, Menstrual Symptom Questionnaire.

After completing the electronic screening and informed consent, eligible participants completed the Baseline questionnaire, which included demographics for the assessment of study diversity and representativeness, and the assessment of PMS-related symptoms through the validated Menstrual Symptom Questionnaire (MSQ) [18], the validated Numerical Pain Rating Scale (NPRS) [19], study-specific questionnaire items assessing self-reported severity and frequency of PMS-related symptoms, frequency of sick days due to menstrual issues, menstrual cycle length, and length of bleeding days. Further questionnaires were completed at the end of Cycle 1 and Cycle 2, which included the assessments completed at Baseline and additional product perception questions evaluating participants’ perceived experience with the test product. At the end of Cycle 2, participants were also asked to report any adverse effects associated with product use. The study assessment schedule is shown in Figure 1.

Questionnaires and scoring

Nine study-specific symptom questionnaire items were developed to evaluate self-reported PMS-related symptoms, including severity of bleeding, fatigue, hormonal acne, cognitive changes, breast tenderness, and gastrointestinal distress. Each item was rated on a five-point Likert scale, where 5 represented the worst outcome, and 1 represented the best outcome; higher scores indicated worse outcomes, and a decrease in score indicated improvement. Three study-specific questions were developed to assess the frequency of sick days due to menstrual issues, menstrual cycle length, and length of bleeding days, assessed with open numerical responses.

Perceived menstrual pain and symptoms were assessed using the validated MSQ. Responses were collected using a five-point Likert scale for each question and converted to numerical values according to the validated scoring system, where 1 (never) represented the best outcome and 5 (always) the worst. Total MSQ scores were calculated for each participant, and the mean scores for all participants were calculated for each time point. A higher composite score indicated a greater number of symptoms.

The NPRS was utilized to assess the intensity of menstrual cramps during the participants’ previous period. The score ranged from 0 (no pain) to 10 (worst pain imaginable), with higher scores indicating more severe pain. The total Numerical Pain Rating at each time point was calculated as the mean score across all participants. A higher composite score indicated a greater level of pain.

Product perception was assessed using study-specific agreement statements regarding perceived product benefit, satisfaction, ease of use, and tolerability. Responses were rated using a five-point Likert scale ranging from “strongly disagree” to “strongly agree.” Product perception outcomes were summarized descriptively as the proportion of participants responding “agree” or “strongly agree”. Agreement by ≥50-69% of participants was regarded as majority participant agreement, while agreement by ≥70% of participants was regarded as strong participant agreement.

Primary and secondary objectives

The primary objective was to evaluate the changes in PMS-related symptom frequency and severity during use of the test product over two menstrual cycles. The principal outcome measure was change in Menstrual Symptom Questionnaire (MSQ) score from Baseline to Cycle 1 and Cycle 2. Additional exploratory outcomes related to the primary objective included menstrual cramping severity assessed using the Numerical Pain Rating Scale (NPRS), frequency of sick days due to menstrual issues, menstrual cycle length, length of bleeding, and nine study-specific Likert-type items assessing menstrual heaviness, bleed-through frequency, fatigue, productivity, breast tenderness, digestive upset, hormonal acne or skin concerns, and cognitive symptoms. These outcomes were assessed at Baseline, Cycle 1, and Cycle 2.

The secondary objective was to assess participants’ perceptions of the product’s effects on their menstrual cycle experience and satisfaction with the product. Secondary endpoints were assessed at the end of Cycle 1 and Cycle 2 using study-specific agreement-based questionnaire items evaluating perceived changes in bloating, menstrual and premenstrual pain, cramping, energy, mood, skin appearance, sleep, breast tenderness, libido, digestion, productivity, menstrual flow, and social interactions, as well as overall product satisfaction.

Data quality and protocol deviations

Data quality was assessed using a quality-control question within the electronic questionnaire. Participants who failed the quality-check question were excluded from the analyses for that time point only; data from the same participant at other valid time points were retained. No imputation was performed for excluded or missing observations, and analyses were conducted using the available data at each time point. One participant was excluded for data quality reasons at the end of Cycle 1 and one at the end of Cycle 2.

Statistical analysis

Demographics were summarized using descriptive statistics. Continuous variables were reported as mean ± standard deviation (SD). For the nine study-specific symptom questionnaire items, Likert-scale responses were converted to numerical scores ranging from 1 to 5 and analyzed as repeated measures outcomes. Data were checked for normality using the D'Agostino & Pearson test and analyzed using a two-way ANOVA test with Dunnett's multiple comparisons test. MSQ scores were calculated according to the validated scoring guidelines. Numerical pain scores, frequency of sick days due to menstrual issues, menstrual cycle length, and length of bleeding days were calculated as the average across participants for each time point. These data were checked for normality using the D'Agostino & Pearson test and analyzed using a Kruskal-Wallis test with Dunn’s multiple comparisons test. All statistical analyses were performed in GraphPad Prism 10.6 (GraphPad Software, LLC, San Diego, California), with the significance level set at p < 0.05. For product-specific questions not evaluated at Baseline, results were presented as the percentage of subjects reporting each answer.

## Results

Demographics and participant characteristics

A total of 40 participants were enrolled in the study. All participants completed the questionnaires across two menstrual cycles. However, at the end of Cycle 1, the data from one participant were excluded due to failure to answer the quality-check question. Another participant's data were also excluded from Cycle 2 for this reason. The mean age of the subjects at Baseline was 36 years ± 6.67 (Table 2).

**Table 2:** Demographics and participant characteristics at baseline. Data are self-reported and presented as mean ± standard deviation (SD) for continuous variables and n (%) for categorical variables.

Demographic characteristic	Total sample (N = 40)
Age, years, mean ± SD	36 ± 6.67
Weight, pounds, mean ± SD	165 ± 41.9
Height, inches, mean ± SD	64 ± 2.38
Sex, N (%)	
Female	40 (100%)
Male	0 (0%)
Race, N (%)	
American Indian or Alaska Native	1 (2.5%)
Asian	0 (0%)
Black or African American	3 (7.5%)
More than one race	0 (0%)
Other	2 (5%)
White	33 (82.5 %)
Prefer not to answer	1 (2.5%)
Ethnicity, N (%)	
Hispanic or Latino	6 (15%)
Not Hispanic or Latino	34 (85%)
Prefer not to answer	0 (0%)

MSQ, pain score, and sick day changes in PMS-related symptom severity

Participants completed the validated MSQ questionnaire and the NPRS for menstrual cramping and reported the frequency of sick days due to menstrual issues at Baseline, the end of Cycle 1, and the end of Cycle 2 (Table 3). By the end of Cycle 2, the MSQ score had significantly decreased, with a 16.62% decrease compared to Baseline. Additionally, menstrual cramping pain, evaluated using the NPRS, had significantly decreased, with a 24.49% decrease compared to Baseline. Results demonstrated that the number of sick days due to menstrual issues significantly decreased by the end of Cycle 2, with a 73.86% decrease compared to Baseline. 

**Table 3:** Changes in Menstrual Symptom Questionnaire (MSQ), Numerical Pain Rating for menstrual pain, and sick days. Data are presented as mean ± standard deviation (SD). A decrease in score indicates an improvement. p-values compare each post-Baseline time point with Baseline. p≤0.05 was considered statistically significant. Statistical significance was given by the Kruskal-Wallis test with Dunn’s multiple comparisons test. ns, p>0.05; **, p≤0.01; ***, p≤0.001; ****, p≤0.0001.

Question	Baseline (N = 40)	Cycle 1 (N = 39)		Cycle 2 (N = 39)		Kruskal-Wallis, H(df), p-value
	Mean ± SD	Mean ± SD	p-value	Mean ± SD	p-value	
MSQ score	3.61 ± 0.54	3.36 ± 0.49	0.154 (ns)	3.01 ± 0.68	<0.0001 (****)	(H(2)=17.08), 0.0002
Numerical Pain Rating for menstrual cramping	6.90 ± 1.45	6.26 ± 1.85	0.233 (ns)	5.21 ± 1.87	0.0003 (***)	(H(2)=14.68), 0.0006
Number of sick days	0.88 ± 1.57	0.53 ± 1.43	0.102 (ns)	0.23 ± 0.74	0.01 (**)	(H(2)=8.25), 0.0162

Changes in the menstrual cycle and bleeding duration

Changes in menstrual cycle length and bleeding duration during use of the Seed Cycling Capsules were evaluated at Baseline and at the end of Cycle 1 and Cycle 2 (Table 4). By the end of Cycle 1, the menstrual cycle length had significantly reduced, with a 24.10% decrease compared to Baseline. However, no significant changes in menstrual cycle length were observed by the end of Cycle 2. There were no significant changes in the length of bleeding by the end of Cycle 1 and Cycle 2 compared to Baseline.

**Table 4:** Changes in menstrual cycle and bleeding duration. Data are presented as mean ± standard deviation (SD). p-values compare each post-Baseline time point with Baseline. p≤0.05 was considered statistically significant. Statistical significance was given by Kruskal-Wallis test with Dunn’s multiple comparisons test. ns, p>0.05; **, p≤0.01.

Question	Baseline (N = 40)	Cycle 1 (N = 39)		Cycle 2 (N = 39)		Kruskal-Wallis, H(df), *p*-value
	Mean ± SD	Mean ± SD	p-value	Mean ± SD	p-value	
Menstrual cycle length (Days)	28.38 ± 2.12	21.54 ± 10.44	0.0036 (**)	24.67 ± 8.29	0.105 (ns)	(H(2)=9.97), 0.0068
Length of bleeding (Days)	5.81 ± 1.27	5.51 ± 1.37	0.718 (ns)	5.31 ± 0.74	0.192 (ns)	(H(2)=2.78), 0.249

Trends in PMS-related symptom outcomes

Changes in the severity and frequency of PMS-related symptoms during use of the Seed Cycling Capsules were evaluated using nine PMS-related symptom parameters, including heaviness of period, bleeding through a tampon or sanitary towel, fatigue frequency and severity, PMS-related symptoms interfering with productivity, breast tenderness, digestive upset, hormonal acne or skin concerns, and brain fog. The parameters were evaluated at Baseline and at the end of Cycle 1 and Cycle 2.

By the end of Cycle 1, there was a significant reduction in how often participants reported digestive upset, such as bloating, constipation, upset stomach, diarrhea, gas, or indigestion, with a 13.08% decrease compared to Baseline (Table 5).

By the end of Cycle 2, all nine PMS-related symptom parameters were significantly reduced compared to Baseline (Table 5). The most notable improvements included a 35.32% decrease in how often PMS-related symptoms interfered with productivity, a 30.51% decrease in how often participants experienced digestive upset, a 29.76% decrease in how often they experienced breast tenderness, a 29.70% decrease in the severity of hormonal acne or skin concerns during their period, and a 27.89% decrease in the severity of fatigue during their period.

**Table 5:** Changes in premenstrual syndrome (PMS)-related symptom parameters. Data are presented as mean ± standard deviation (SD). A decrease in score indicates a reduction in the severity and frequency of symptoms. p-values compare each post-Baseline time point with Baseline. p≤0.05 was considered statistically significant. Statistical significance was given by a two-way ANOVA test with Dunnett's multiple comparisons test (F (2, 1035) = 97.91, p<0.0001). ns, p>0.05; *, p≤0.05; **, p≤0.01; ***, p≤0.001; ****, p<0.0001.

Parameter	Baseline (N = 40)	Cycle 1 (N = 39)		Cycle 2 (N = 39)	
	Mean ± SD	Mean ± SD	p-value	Mean ± SD	p-value
Menstrual heaviness	3.93 ± 0.83	3.72 ± 0.79	0.518 (ns)	3.33 ± 0.81	0.0102 (*)
Bleed-through frequency	3.25 ± 1.28	2.82 ± 1.41	0.0783 (ns)	2.36 ± 1.11	<0.0001 (****)
Fatigue frequency	3.90 ± 0.63	3.90 ± 0.64	0.999 (ns)	3.26 ± 0.64	0.0047 (**)
Fatigue severity	3.80 ± 0.69	3.62 ± 0.88	0.589 (ns)	2.74 ± 0.82	<0.0001 (****)
Productivity impact	3.85 ± 0.86	3.44 ± 1.02	0.0923 (ns)	2.49 ± 0.94	<0.0001 (****)
Breast tenderness	3.73 ± 1.09	3.54 ± 1.19	0.583 (ns)	2.62 ± 0.94	<0.0001 (****)
Digestive upset	4.13 ± 0.79	3.59 ± 0.99	0.0221 (*)	2.87 ± 0.92	<0.0001 (****)
Skin concern severity	3.03 ± 0.89	2.69 ± 1.00	0.203 (ns)	2.13 ± 0.77	<0.0001 (****)
Brain fog severity	3.20 ± 1.02	3.03 ± 1.04	0.623 (ns)	2.36 ± 0.93	0.0002 (***)

Product perception and satisfaction

Participants completed responses to 15 questions at the end of Cycle 1 and 15 questions at the end of Cycle 2 that provided insight into their perceptions of the test product. By the end of Cycle 1, one of the 15 parameters demonstrated ≥50-69% participant agreement, which included perceptions of improved mood stability (N = 20, 51.28%) (Table 6).

By the end of Cycle 2, nine of the 15 parameters demonstrated ≥50-69% participant agreement, while one of the 15 demonstrated strong participant agreement (≥70%). The greatest agreement scores were observed for perceptions related to improved menstrual pain severity (N = 28, 71.79%), bloating severity (N = 26, 66.67%), cramp severity (N = 26, 66.67%), mood stability (N = 24, 61.54%), skin clarity (N = 24, 61.54%), and sleep (N = 24, 61.54%) (Table 6). By the end of Cycle 2, 36 (92.31%) participants reported that they took the test product every day, while 3 (7.69%) participants reported that they took the test product most days.

**Table 6:** Participant-reported product perception and satisfaction outcomes. Data are presented as the percentage of participants who selected “agree” or “strongly agree” for each statement.

Question	Combined Agree Score	
	Cycle 1 (N = 39)	Cycle 2 (N = 39)
My bloating is less severe since using this product	15 (38.46%)	26 (66.67%)
My menstrual/pre-menstrual pain is less severe since using this product	17 (43.59%)	28 (71.79%)
My cramps are less severe since using the product	16 (41.03%)	26 (66.67%)
I have more energy since using this product	19 (48.72%)	23 (58.97%)
My mood has been more stable since using this product	20 (51.28%)	24 (61.54%)
I have had fewer breakouts since using this product	14 (35.90%)	23 (58.97%)
My skin looks clearer since using the product	12 (30.77%)	24 (61.54%)
I can sleep better since using this product	13 (33.33%)	24 (61.54%)
I have less breast tenderness since using this product	17 (43.59%)	21 (53.85%)
My libido has increased since using this product	12 (30.77%)	19 (48.72%)
My digestion has improved since using this product	14 (35.90%)	16 (41.03%)
My productivity has improved since using this product	14 (35.90%)	17 (43.59%)
My period is lighter since using this product	16 (41.03%)	23 (58.97%)
The quality of my social interactions during my periods has improved since using this product	11 (28.21%)	18 (46.15%)
I have fewer sick days related to my menstrual cycle since taking this product	8 (20.51%)	15 (38.46%)

Adverse events and tolerability

No serious adverse events were reported throughout the study, and the product was well tolerated overall. By the end of Cycle 2, one participant reported light spotting on Day 14, two participants reported digestive upset, one participant reported heartburn, one participant reported irritability and breakouts, and one participant reported that their menstrual cycle symptoms were more sporadic. One participant also reported feeling emotional during the first cycle and that their second cycle was shorter, lighter, and came earlier than expected.

## Discussion

This study evaluated changes in the severity and frequency of PMS-related symptoms associated with use of the test product over two menstrual cycles, using a combination of participant-reported outcomes and standardized assessments of PMS-related symptoms. MSQ score, menstrual cramping assessed by the validated NPRS, and the number of sick days due to menstrual issues demonstrated significant improvements by the end of Cycle 2. Findings from the evaluation of menstrual cycle length indicated that menstrual cycle length was reduced relative to Baseline at Cycle 1 during product use; however, this effect was not sustained by the end of Cycle 2. Additionally, there were no significant differences in the length of bleeding. By the end of Cycle 2, all nine study-specific parameters had significantly improved, and participant perception data demonstrated that most participants agreed that their menstrual or premenstrual pain and cramps were less severe, their skin was clearer, they slept better, they had less bloating and breast tenderness, and their periods were lighter, aligning with the significant results obtained across the standardized assessments.

The observed changes during use of the test product may be explained by several biological mechanisms related to seed intake. The nutritional content of the tested seeds, such as lignans, omega-3, phytoestrogens, vitamin E, selenium, and fiber, has been demonstrated to impact hormonal balance through phytoestrogenic properties, modulate enzyme activity, and integrate into hormone synthesis pathways [3,4,6,11], in addition to possessing antioxidant and anti-inflammatory properties [14]. As such, the results of this study corroborate those found in related PMS research. Intake of lignans, omega-3, zinc, phytoestrogens, and vitamin E has been associated with improved PMS symptoms among diverse populations, including those with PMS [20-24].

The present study reports a 16.62% reduction in the MSQ score upon intake of the test product, suggesting a reduction in participant-perceived PMS-related symptoms. Improvements in Premenstrual Symptoms Questionnaire (PSQ) scores have also been observed in women with PMS consuming chasteberry [16], with intake of sesame seeds, flaxseeds, pumpkin seeds, and sunflower seeds likewise improving menstrual symptoms in women with dysmenorrhea and PMOS [4]. However, because chasteberry was administered only during days 15 to 28 (i.e., the luteal phase), its effect may not be comparable to the continuous daily administration used in many prior studies [15,16,25]. Limited exposure may have reduced the opportunity for cumulative endocrine modulation, including dopaminergic effects on prolactin and related luteal phase regulation. Nonetheless, because of effects associated with dopaminergic suppression of prolactin and possible support of luteal phase hormonal regulation, its inclusion may have provided a clear mechanism contributing to adverse symptom reduction. Chasteberry has also been hypothesized to influence broader hypothalamic-pituitary signaling, including the HPA axis [10]. Although this remains insufficiently substantiated in human studies, and the effects of an individual component cannot be separated from the multiseed formulation of the test product, these mechanisms could potentially be relevant to the observed symptom changes. Likewise, while one study indicates fiber has not been found to have an effect on women with self-reported PMS [26], studies of women who experience menstrual pain and PMS-related symptoms show that fiber supplementation can improve symptoms associated with inflammation [27,28], an effect that may account for the improvements in menstrual cramping and the number of sick days due to menstrual issues observed in this study.

Alteration of menstrual cycle duration is not the intended purpose of seed cycling. However, menstrual cycle length was significantly lower at Cycle 1 than at Baseline during use of ZEST, consisting of flaxseed and pumpkin seed, and ZEN, consisting of sesame seed, sunflower seed, chasteberry, and psyllium. The same effect was not observed during Cycle 2, suggesting that the change may not reflect a consistent effect associated with product use. Given the short study duration and expected intercycle variability in menstrual cycle length, this isolated finding may reflect normal cycle-to-cycle variation, regression to the mean, or baseline variability rather than a reproducible effect of seed cycling. Although changes in menstrual cycle length have been reported following flaxseed intake in vivo and in women with PMOS [4,6,29], the absence of a similar effect during Cycle 2 suggests that the observed decrease should be interpreted cautiously.

This study design has a number of strengths. It allowed for repeated measurements across two menstrual cycles. The intervention reflected consumer-relevant, real-world product use, and the outcomes assessed were relevant to adults seeking support for PMS-related symptoms. In addition, the product showed good short-term tolerability, with no serious adverse effects reported. Participant perception outcomes also indicated high acceptability and a willingness to continue using the product.

Several limitations should, however, be taken into account when interpreting the findings. The principal limitation is that the study used a single-arm, open-label design and did not include a placebo control, limiting the ability to establish causality and increasing the possibility of expectancy or placebo effects, which can produce small but measurable improvements in self-reported outcomes [30]. Furthermore, all study outcomes were based on self-reported questionnaire responses, including study-specific questionnaires that have not been formally validated. The study was also relatively short, carried out over two menstrual cycles, and biomarkers of hormones (i.e., estrogen), the gut microbiome, and inflammation were not assessed throughout the study. This makes it difficult to determine whether participants experienced measurable changes in hormonal balance, mood, inflammation, and digestive upset related to the test product or whether the improvements were subjective.

Despite these limitations, the preliminary findings suggest that use of seed cycling with chasteberry is associated with improvements in PMS-related symptoms and may represent a well-tolerated adjunctive management strategy, providing a strong rationale for further investigation. Future studies should include long-term, randomized controlled trials in women diagnosed with PMS, alongside biomarker analyses, to better characterize the potential symptomatic and physiological effects of seed cycling and chasteberry.

## Conclusions

These results suggest that the Two Moons Seed Cycling Capsules with chasteberry may be a well-tolerated option associated with cumulative symptom improvement. The intake of ZEST, containing traditional seed cycling ingredients organic flaxseed and organic pumpkin seed, and ZEN, containing organic sesame seed, organic sunflower seed, chasteberry, and psyllium, was associated with significant improvements in key areas, including MSQ score, menstrual cramping pain, and sick days due to menstrual-related issues, with the most notable improvements being reductions in the frequency of PMS symptoms interfering with productivity or causing bleed-through and in the severity of breast tenderness, fatigue, digestive upset, skin concerns, and brain fog. Participant feedback reflected strong satisfaction with the product, including perceived improvements in premenstrual and menstrual pain, cramping, bloating, and skin clarity. These results suggest that use of the test product is associated with several improvements and may represent a well-tolerated option for women seeking to manage the severity and frequency of PMS-related symptoms. Further research conducted using a randomized controlled design and a larger sample size could help validate and expand upon these preliminary findings.

## Appendices

**Table 7:** Baseline study questionnaire.

Response Options
Group B: Not noticeable, Barely noticeable, Moderate, Distracting, Severe
Group C: Never, Rarely, Sometimes, Often, Always
Group C2: Very light, Light, Moderate, Heavy, Very heavy
Group H2: 1, = No pain, 2, 3, 4, 5, 6, 7, 8, 9, 10 = Worst pain imaginable
Group I: Blank text box
Questions
1. Menstrual Symptom Questionnaire [18] (Response Group: C)
2. Numerical Pain Rating Scale [19] (Response Group H2)
3. Study-Specific Questions
· How many days was your last menstrual cycle (this is usually measured from the first day of your last period to the day before your next period)? (Response Group: I)
· Thinking back to your last period:
· How heavy was your period? (Response Group: C2)
· How many days did your period last, from the time the bleeding began until it completely stopped? (Response Group: I)
· How often did you have bleeding where you would bleed through a tampon or sanitary towel in two hours or less? (Response Group: C)
· How often did you feel fatigued during the last four weeks? (Response Group: C)
· How severe was your fatigue during your period? (Response Group: B)
· How often did you feel that your PMS symptoms interfered with your productivity? (Response Group: C)
· How often did you feel breast tenderness? (Response Group: C)
· How often have you felt digestive upset (bloating, constipation, upset stomach, nausea, diarrhea, gas, indigestion)? (Response Group: C)
· How would you rate the severity of your hormonal acne or skin concerns around your period? (Response Group: B)
· How would you rate the severity of any brain fog that you experienced during your period? (Response Group: B)
· How many sick days did you need to take during your last cycle, due to menstrual issues? [Response group: I)

**Table 8:** Cycle 1 and Cycle 2 study questionnaires.

Response Options
Group B: Not noticeable, Barely noticeable, Moderate, Distracting, Severe
Group C: Never, Rarely, Sometimes, Often, Always
Group C2: Very light, Light, Moderate, Heavy, Very heavy
Group H2: 1, = No pain, 2, 3, 4, 5, 6, 7, 8, 9, 10 = Worst pain imaginable
Group I: Blank text box
Group D: Strongly agree, Agree, Neither agree or disagree, Disagree, Strongly disagree
Questions
1. Menstrual Symptom Questionnaire [18] (Response Group: C)
2. Numerical Pain Rating Scale [19] (Response Group H2)
3. Study-Specific Questions:
· How many days was your last menstrual cycle (this is usually measured from the first day of your last period to the day before your next period)? (Response Group: I)
· Thinking back to this most recent period:
· How heavy was your period? (Response Group: C2)
· How many days did your period last, from the time the bleeding began until it completely stopped? (Response Group: I)
· How often did you feel fatigued during the last four weeks? (Response Group: C)
· How severe was your fatigue during your period? (Response Group: B)
· How often did you feel that your PMS symptoms interfered with your productivity? (Response Group: C)
· How often did you feel breast tenderness? (Response Group: C)
· How often have you felt digestive upset (bloating, constipation, upset stomach, nausea, diarrhea, gas, indigestion)? (Response Group: C)
· How would you rate the severity of your hormonal acne or skin concerns around your period? (Response Group: B)
· How would you rate the severity of any brain fog that you experienced during your period? (Response Group: B)
· How many sick days did you need to take during your last cycle, due to menstrual issues? [Response group: I)
4. How much do you agree or disagree with the following: (Response Group: D)
· My bloating is less severe since using this product
· My menstrual/pre-menstrual pain is less severe since using this product
· My cramps are less severe since using the product
· I have more energy since using this product
· I am answering this survey carefully and paying attention to each question.
· My mood has been more stable since using this product
· I have had fewer breakouts since using this product
· My skin looks clearer since using the product
· I can sleep better since using this product
· I have less breast tenderness since using this product
· My libido has increased since using this product
· My digestion has improved since using this product
· My productivity has improved since using this product
· My period is lighter since using this product.
· The quality of my social interactions during my periods has improved since using this product
· I have fewer sick days related to my menstrual cycle since taking this product.

**Table 9:** Cycle 2 only study questionnaire.

Response Options
Group X: Never, Rarely, Some days, Most days, Every day
Group D: Strongly agree, Agree, Neither agree or disagree, Disagree, Strongly disagree
Group H: Very satisfied, Satisfied, Neither satisfied nor dissatisfied, Dissatisfied, Very dissatisfied
Group I: Blank text box
Questions
· I would like to continue using this product. (Response Group: D)
· I would recommend this product to my family and friends. (Response Group: D)
· How satisfied are you overall with this product? (Response Group: H)
· Did you experience any side effects or adverse events during this study? If yes, please detail here. (Response Group: I)
· How consistently did you take the product as recommended? [Response Group: X]
